# Genetic Characterization of Atypical *Citrobacter freundii*

**DOI:** 10.1371/journal.pone.0074120

**Published:** 2013-09-12

**Authors:** Gabriela Delgado, Valeria Souza, Rosario Morales, René Cerritos, Andrea González-González, José Luis Méndez, Virginia Vázquez, Alejandro Cravioto

**Affiliations:** 1 Departmento de Microbiología y Parasitología, Facultad de Medicina, Universidad Nacional Autónoma de México (UNAM), México City, México; 2 Departamento de Ecología Evolutiva, Instituto de Ecología, Universidad Nacional Autónoma de México (UNAM), México City, México; 3 Departamento de Cirugía Experimental, Facultad de Medicina, Universidad Nacional Autónoma de México (UNAM), México City, México; 4 Instituto Nacional de Pediatría (INP), México City, México; 5 International Vaccine Institute, Seoul, Republic of Korea; University of Birmingham, United Kingdom

## Abstract

The ability of a bacterial population to survive in different niches, as well as in stressful and rapidly changing environmental conditions, depends greatly on its genetic content. To survive such fluctuating conditions, bacteria have evolved different mechanisms to modulate phenotypic variations and related strategies to produce high levels of genetic diversity. Laboratories working in microbiological diagnosis have shown that *Citrobacter freundii* is very versatile in its colony morphology, as well as in its biochemical, antigenic and pathogenic behaviours. This phenotypic versatility has made *C. freundii* difficult to identify and it is frequently confused with both *Salmonella enterica* and *Escherichia coli*. In order to determine the genomic events and to explain the mechanisms involved in this plasticity, six *C. freundii* isolates were selected from a phenotypic variation study. An I-*Ceu*I genomic cleavage map was created and eight housekeeping genes, including 16S rRNA, were sequenced. In general, the results showed a range of both phenotypes and genotypes among the isolates with some revealing a greater similarity to *C. freundii* and some to *S. enterica*, while others were identified as phenotypic and genotypic intermediary states between the two species. The occurrence of these events in natural populations may have important implications for genomic diversification in bacterial evolution, especially when considering bacterial species boundaries. In addition, such events may have a profound impact on medical science in terms of treatment, course and outcomes of infectious diseases, evading the immune response, and understanding host-pathogen interactions.

## Introduction

The correct identification and determination of bacterial species plays an important role in the diagnosis of infectious diseases, food safety, and epidemiology. Human response to bacterial infection depends on the biological properties of the infecting microorganism, as well as on the natural variation of the species and its potential to change [1]. These variations are the adaptive responses by which bacteria undergo frequent and reversible phenotypic changes resulting from genetic modifications in their genomes [2]. Despite the different molecular mechanisms that mediate and regulate phenotypic variation, these related gene regulatory systems are not sufficient to allow bacteria to manage environmental challenges. To survive, bacteria have evolved assorted strategies to produce high levels of genetic diversity through complex combinations and reshuffling of genetic information [2], [3]. This heterogenic response enables a bacterium to utilize different niches within an ecosystem, and even to increase the overall fitness of the species, all of which are reflected in life-style differences and physiological versatility [4], [5].

Although the chromosomal organization of related bacteria is conserved, a considerable genomic variability and diversification between isolates of the same species has been observed as a result of distinct molecular events involved in the adaptive evolution of the genomes [5]. Point mutations, homologous recombination, deletions, duplications, inversions, and horizontal gene transfer contribute to this variation, and consequently to genome diversification.

The dynamic nature of these bacterial gene pools, and in particular the ability of genes to move between non-related bacterial groups, has complicated the concept of species coherence for such microorganisms. In this respect, species boundary for bacteria continues to be controversial, especially in terms of the mechanisms of bacterial speciation and maintenance in the face of frequent genetic interchange [6]. Some species of the genus *Citrobacter* present this type of behaviour [7] and it is this fact that has made *C. freundii* difficult to identify, since the species is similar to other members of the Enterobacteriaceae family. Although the majority of isolates of *C. freundii* are frequently identified as belonging to typical strains, atypical biotypes also exist, differing from typical biotypes in that they have variable culture, biochemical and antigenic characteristics that resemble those of the genus *Salmonella* and *Escherichia coli* [8]. This plasticity has prevented a definitive classification of *C. freundii* and has created a taxonomic problem that has been under discussion for many years, with classification and nomenclature varying over time according to the author concerned [9]–[11].

In addition, the *Citrobacter* genus has a large number of somatic (O) and flagella (H) antigens typically found in other species of the Enterobacteriaceae family, in particularly in *Salmonella* [8], [9]. Furthermore, studies of lipopolysaccharide (LPS) structures in enteric bacteria have highlighted the structural mimicry of the somatic antigen of *C. freundii* [12]–[16].

Other studies of *C. freundii* have identified virulence genes that are homologous or identical to those described in *E. coli* pathotypes and *Salmonella* [16]–[21]. DNA-DNA hybridization [11], [22] used to assess divergence in species of *Citrobacter* and *Citrobacter*-like strains has also revealed substantial heterogeneity within strains identified as *C. freundii*. Between 1960 and 1990, various laboratories [23]–[28] began to isolate strains that could be treated as atypical *C. freundii* or as strains of lactose-positive *Salmonella enterica* associated with gastroenteritis. These findings highlighted the close similarity between some *C. freundii* strains and *Salmonella enterica* and the confusion that may arise in differentiating between these two genera.

The availability of a significant number of bacterial genomes has enabled the phylogenetic relationship among bacterial populations to be assessed. Such detailed research will allow a better definition of the different isolates, pathotypes and even species [29], [30].

A number of recent studies have started to shed light on the dynamic chromosomal organization, genetic content and genetic diversity of *Citrobacter* [29]–[31]. The aim of the current study was to detect the genomic changes presented in atypical *C. freundii* isolates during biochemical phenotype variation, and to discuss the implications of such changes with regard to their possible impact on the boundaries of the species.

## Materials and Methods

### Background of the Bacterial Isolates

In August 1985, a longitudinal study was initiated to look at the agents associated with acute diarrhoea in a group of 75 children living in a rural village in México [32], [33].

Weekly faecal samples were obtained from each child in the study over a two-year period. Isolation, identification and classification of the enteric pathogens were carried out as described by Edwards and Ewing [9], [33]. Strains identified as typical and atypical *C. freundii* were further used for this study. Atypical *C. freundii* differ mainly in their capacity to ferment lactose, decarboxylase lysine and utilize potassium cyanide (KCN). All of the isolated strains were labelled and stored on sealed Dorset egg slants, in a temperature-controlled environment.

**Figure 1:**
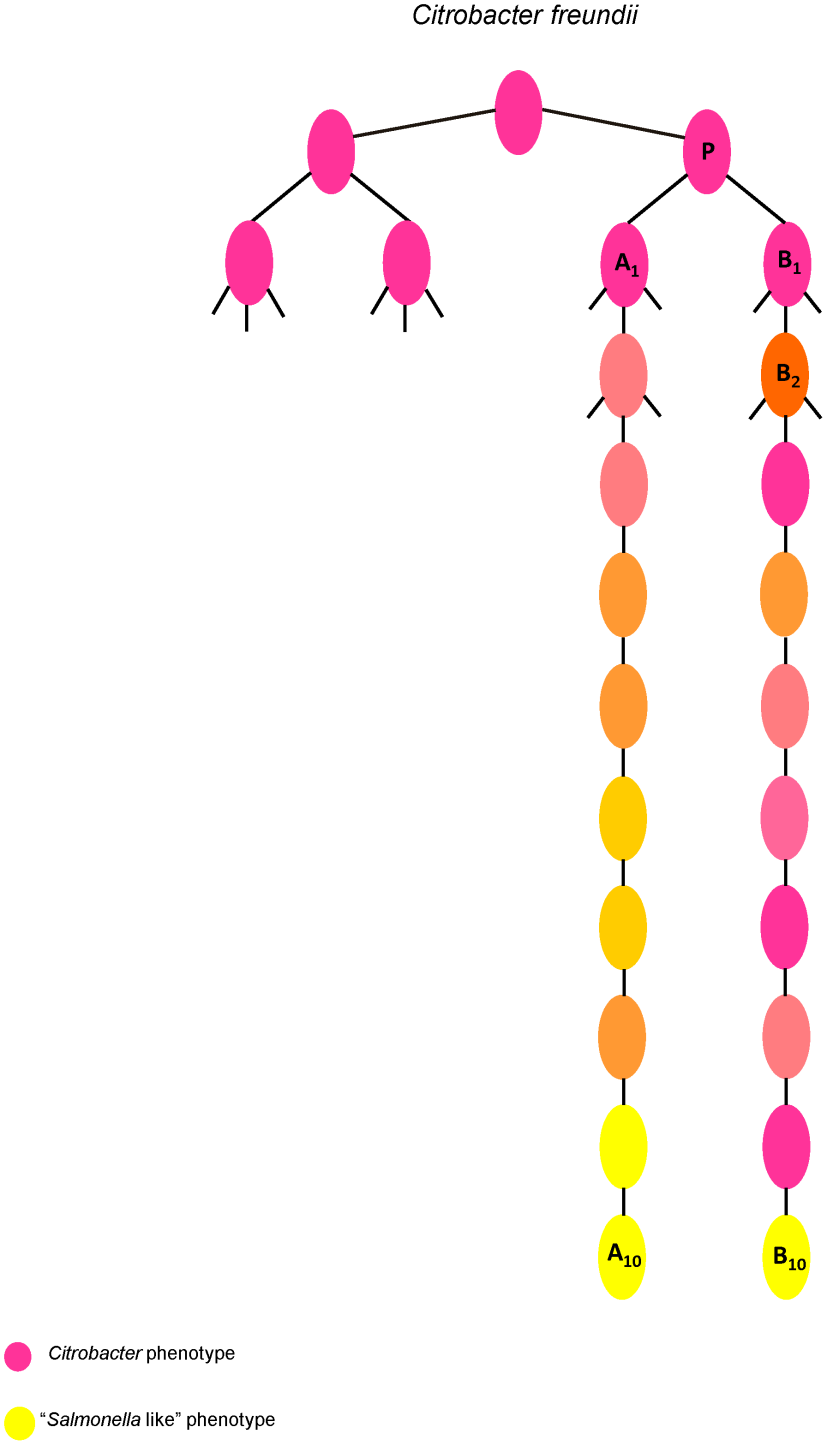
Bacterial lineages derived from the phenotypic diversification study. Shows the lineages A and B derived from the *Citrobacter freundii* FMU108327/P, each dot represents a selected colony. The pink dots indicate a typical *C. freundii* biochemical phenotype; yellow dots indicate a “*Salmonella*-like” biochemical phenotype; the dots in gradient of pink to yellow represent the intermediates biochemical phenotypes. The solid lines indicate the number of colonies selected after each selective round. The parental strain was *Citrobacter freundii* FMU108327/P, and after the first selection two descendants were generated, which were used to found the next selection, which led to six descendants (in both lineages) being selected, and all of these isolates were used to found the 3^th^ selection round. This procedure was repeated throughout the 10 selections. The numbered dots P, A_1_, A_10_, B_1,_ B_2_ and B_10_ indicate the isolates selected for this study.

In late 1989, V. Vázquez and A. Cravioto conducted a phenotypic variation study (data not published) with the *C. freundii* strains that had been isolated from the previously described longitudinal survey [32], [33]. The bacteriological methods used in the study included the repeated streaking of single colonies on *Salmonella-Shigella* agar (SS agar) and their cultivation to avoid the possibility of both contamination and mixing of the microorganisms, all of which led to the selection of pure colonies. A strain initially identified as atypical *C. freundii* was selected for further studies by performing subsequent passages under identical conditions. Briefly, the atypical *C. freundii* isolate was grown overnight in a sterile liquid media. The resulting culture was streaked onto SS agar plates and incubated overnight at 37°C. Following incubation, all of the single colonies showing morphologic differences were selected, and then, each one were grown in different biochemical test media. The biochemical profile of each isolate were observed and scored (Figure 1, Table 1). Cells from each single colony were used to found the next selection round and so on (Figure 1). The methods of cultivation, isolation and environment conditions were exactly the same of those of the previous round. This procedure was repeated throughout all 10 selective rounds (Figure 1). This resulted in the creation of a large number of descendants, which could then be used to determine the phenotypic and genomic characteristics that made these isolates different from typical *C. freundii* strains (supporting information). Single colonies from these cultures were selected using a stereoscopic microscope [34]. The selected colonies were characterized using forty standard biochemical tests as described by Ewing (Table 1).

**Table 1:** Biochemical test of *Citrobacter freundii* isolates.

Test	*C. freundii*NCTC E9750	FMU108327/P	FMU108327/A_1_	FMU108327/A_10_	FMU108327/B_1_	FMU108327/B_2_	FMU108327/B_10_	*Salmonella* *enterica*
catalase	+	+	+	+	+	+	+	+
NO_3_ - NO_2_	+	–	+	+	+	+	+	+
H_2_S (KIA)	+	+	+	+_w_	+	+_w_	+	+
KIA	A/A	A/A	A/A	Alk/A	A/A	Alk/A	Alk/A	Alk/A
Lactose	V+	+	+	–	+	–	–	–
Sucrose	+	+	+	–	+	–	–	–
Adonitol	–	–	–	–	–	–	–	–
Cellobiose	+^2nd day^	+^2nd day^	+^2nd day^	–	+^2nd day^	–	+	–
Dulcitol	–	–	–	+	+	+	+	+
Inositol	–	–	–	–	–	–	–	–
Raffinose	+	+	+^2nd day^	–	+	+	–	–
Salicine	–	–	–	–	–	–	–	–
D-Sorbitol	+	+	+	+	+	+	+	+
D-Xylose	+	+	+	+	+	+	+	+
Indol	–	–	–	–	–	–	–	–
Methyl red	+	+	+	+	+	+	+	+
VP^1^	–	–	–	–	–	–	–	–
Citrate^2^	+	+	+	+	+	+	+	+
Arginine^3^	+	+	+	+	+	+	+	+
Lysine^4^	–^4day^	–^4day^	–^4day^	+	+_w_	+	+^3day^	+
Ornithine^4^	+	–	–	+	+_w_	+	+	+
Urea broth	+^2nd day^	+	+	–	–	–	–	–
Phenylalaniine^5^	+_w_	+_w_	+_w_	–	+_w_	+_w_	–	–
Gluconate	–	–	–	–	–	–	–	–
KCN broth^6^	+	+	+	+	+	+	+_w_	–
Malonate^7^	–	–	–	–	–	–	–	–
ONPG^8^	+	+	+	–	+	–	–	–
Motility	+	+	+	+	+	+	+	+

Biochemical reaction of *C. freundii* and *S. enterica* as they are currently defined and the biochemical reactions of the *C. freundii* isolates from the phenotypic diversification study.

+_w_ weak reaction if positive.

Approximately 15,500 isolates were obtained in these sequential passages, of which 23% presented the typical biochemical phenotype of *C. freundii*, 14% showed biochemical patterns that resembled *Salmonella* spp., and the remaining 63% presented variable biochemical phenotypes, making precise identification of the atypical isolates difficult using this set of tests (Figure 1, Table 1).

All of the isolates obtained from the Vázquez and Cravioto study were labelled and stored on sealed Dorset egg slants in a temperature-controlled room that housed the culture collection at the Faculty of Medicine UNAM (FMU) of the Universidad Nacional Autónoma de México (UNAM).

### Bacterial Strains

In the present study, six isolates derived from the Vázquez and Cravioto study were selected for further study: FMU108327 (P), and its derived isolates FMU108327/A_1_ (A_1_), FMU108327/A_10_ (A_10_), FMU108327/B_1_ (B_1_), FMU108327/B_2_ (B_2_) and FMU108327/B_10_ (B_10_) (Figure 1). These isolates were selected according to the changes found in their biochemical phenotype. To dispel any doubts that the ensuing results were due to contamination or careless mix-up when the colonies were picked from the agar plates, the purity of the selected isolates were verified using two methods: 1) streaking on *Salmonella-Shigella* agar (SS agar) to ensure the presence of isolated colonies followed by overnight incubation at 37°C. A colony of each isolate was then selected with the aid of a stereoscopic microscope in a sterile area [8], [34] and standard biochemical tests were applied to confirm the original results [9] (Table 1); 2) the derived isolated colonies were diluted and vortexed in 0.1 ml of 0.01% Tween 40 and 0.1 M MgSO_4_ (V/V) and streaked onto SS agar to recheck the purity of the colony [35], [36].

Strains of *Salmonella enterica* serovar Typhimurium LT2 ATCC 700720 obtained from the Enteric Pathogen Reference Laboratory, Public Health Department of the Facultad de Medicina, UNAM, and *C. freundii* E9750 NCTC from the NCTC culture collection in London, UK, were used as reference strains. For subsequent analyses, bacteria were routinely cultured on Luria-Bertani (LB) medium and incubated overnight at 37°C. For the Pulsed Field Gel Electrophoresis (PFGE), only two isolates were used, *C. freundii* FMU108327/P and FMU108327/A_10_. All isolates deriving from this process were labelled and stored at −70°C as glycerol stocks.

### I-*Ceu*I Cleavage Map of the FMU108327/P and 108327/A_10_ Genome

Pulsed field gel electrophoresis (PFGE) was used to construct the ribosome skeleton of the chromosome from the FMU108327/P and FMU108327/A_10_ isolates. Bacterial genomic DNA from *S.* Typhimurium LT2 cleaved with I-*Ceu*I was used as a molecular-weight marker. Since the size of these identifying fragments and gene markers had been determined previously [37], [38], their inclusion improved the precision of chromosome size estimation.

### Preparation and Digestion of Genomic DNA in Agarose Blocks

Genomic DNA of each strain was prepared in agarose blocks using a previously described method [37] with some modifications. Briefly, a second incubation in proteinase K solution was carried out to increase the purity of the DNA. Agarose blocks were pre-incubated in 1× buffer for 30 min at 37°C and then incubated overnight with 100 µl of fresh 1× buffer containing 4 U of I-*Ceu*I (New England Biolabs) at 37°C to digest DNA in the blocks.

### Pulsed Field Gel Electrophoresis (PFGE)

I-*Ceu*I (hereafter called *Ceu*I) fragments were separated by a CHEF Mapper electrophoresis system (Bio-Rad). Electrophoresis was performed on a 1% agarose (Seakem Gold agarose, BioWhittaker Molecular Applications) gel and 0.5× TBE (90 mM Tris, 90 mM boric acid, 2 mM EDTA, pH8) buffer at 12°C. Three stage conditions were applied to the electrophoresis process: stage 1: pulse time ramped from 50 s to 2 min over 21 hrs**;** stage 2: pulse time ramped from 20 s to 1 min 20 s over 10 hrs; and stage 3: pulse time ramped from 3 s to 12 s over 7 hrs at 4 V/cm. The DNA fragments were then transferred onto N+nylon membrane (Amersham Biosciences) by Southern blotting as described previously [39].

### Preparation of DNA Probe and Hybridization

**Figure 2:**
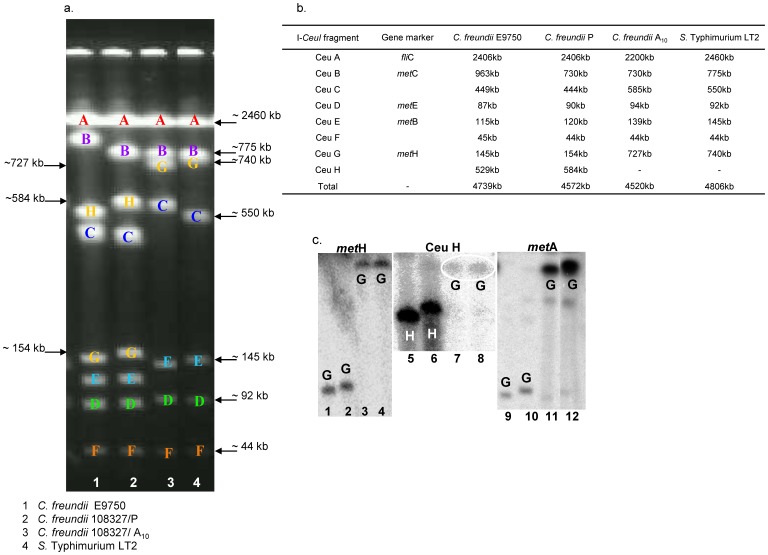
Pulsed-field gel electrophoresis profiles of l-*Ceu*I restriction fragments from *C. freundii* isolates. a) Pulsed-field gel shows *Ceu*I profiles: line 1, *C. freundii* E9750 NCTC; lane 2, FMU108327/P; lane 3, FMU108327/A_10_ and lane 4; *S.* Typhimurium LT2 ATCC 700720. The results of Southern blotting analysis appear with capital letters. These indicate the Ceús individual fragments of *C. freundii* corresponding to those of *S*. Typhimurium LT2; b) chromosome sizes and molecular weights of individual *Ceu*I fragments. It also shows the location of every marker gene according to its correspondence in each *Ceu*I fragment. The *Ceu*I-C and *Ceu*I-F fragments of *C. freundii* were determined by probing the isolated DNA from *S.* Typhimurium LT2 *Ceu*I-C and *Ceu*I-F fragments; c) the gel from panel a were blotted to N+nylon membranes and probed as follows: lanes 1–4 *met*H (Table S1); lanes 5–8 DNA from *C. freundii* fragment *Ceu*I-H and lanes 9–12, *met*A. *Ceu*I fragments identified by the probes are indicated inside the images with capital letters.

The individual fragments of FMU108327/P and FMU108327/A_10_ corresponding to those of *S.* Typhimurium LT2 were determined by probing the Southern blotting membranes with polymerase chain reaction (PCR) products from *S.* Typhimurium LT2 genes whose location in the chromosome is already known (Figure 2). The genes *metB*, *metC*, *metE*, *metH*, *fliC* [40] and *gnd* were selected according to Liu *et al.* [37]. These were used as marker genes to identify the individual *Ceu*I fragments (*metB* on *Ceu*I fragment E, *metC* on *Ceu*I-B, *metE* on *Ceu*I-D and *metH* on *Ceu*I-G, *fliC* and *gnd* on *Ceu*I-A). The primer sets used to amplify the *metB*, *metC*, *metE*, *metH* and *gnd* genes were designed in the laboratory (Table S1) from previously sequenced *S.* Typhimurium LT2 genome (AE006468) [41] using the Primer Select program of the DNASTAR Lasergene 7 package (DNASTAR, Inc., Madison, WI). Correspondence of *Citrobacter* fragments *Ceu*I-C and *Ceu*I-F were determined by probing the isolated DNA from *S.* Typhimurium LT2 *Ceu*I fragments C and F. The PCR products and restriction fragments *Ceu*I-C and F were purified and then ^32^P-labelled using the random primer method of the Megaprime DNA labelling system (Amersham Biosciences). The membrane was incubated in 10 ml hybridization solution (Rapid-hyb buffer) to which 1× Denhardt’s solution and 0.2 mg/ml of denatured sheared salmon sperm were added. Incubation was carried out at 60°C with constant, gentle shaking for 3 h. The labelled probe was then added to fresh hybridization solution and hybridization was carried out overnight at 60°C with constant and gentle shaking. The membrane was exposed to X-ray film after being washed at high astringency (64°C).

### PCR and Hybridization to Determine *rrn* Arrangement

The relative chromosomal position of each *rrn* operon was determined by PCR amplification of the genes adjacent to each operon. These genes are at the opposite ends of each *Ceu*I fragment. The sequences of the used primer and their locations relative to proximal *rrn* operons are shown in Table S1. Primers were designed in the laboratory based on a *S.* Typhimurium LT2 sequence. The PCR products were purified and then ^32^P-labelled before being probed onto Southern blots of *Ceu*I digested DNA.

### Chromosomal DNA Isolation for PCR

Chromosomal DNA was isolated from stationary-phase bacteria taken from cultures grown overnight. DNA was purified by miniprep of bacterial genomic DNA as described previously by Murray and Thompson [42].

### Detection of *cadA* and *lacY* Genes

The adaptive genes, *cadA* and *lacY* that encode lysine decarboxylase and lactose permease, respectively, were amplified in order to detect their presence in the *C. freundii* E9750 strain, the atypical FMU108327/P isolate and its derivatives, since these genes enhance bacterial fitness and confer survival advantage. The primers used to amplify these gene fragments were designed in the laboratory (Table S1). The PrimerSelect program of the DNASTAR Lasergene 7 package (DNASTAR, Inc., Madison, WI) was used to design *cadA* primers from previously sequenced *E. coli* (U00096, AE005174) [43], [44] and *S. enterica* genomes (AE006468, AE014613, AL513382) [41], [45], [46]. The *lacY* primer set was designed from the sequenced *Citrobacter freundii lacY* gene (U13675) [47]. Thermal cycling conditions for both genes were as follows: an initial denaturation cycle at 94°C for 5 min, followed by 30 cycles at 94°C for 1 min, annealing temperature (Table S1) for 1 min and 72°C for 1 min with a final cycle of 72°C for 7 min.

### Amplification and Sequencing Housekeeping Genes and 16S rRNA Gene

Eight housekeeping gene fragments and the 16S rRNA gene were amplified and sequenced for the *C. freundii* E9750 strain, the atypical FMU108327/P and its derivative isolates. The eight genes referred to here were: *adk* (U00096), *aph* (U00096, AE005174, AE006468, and AE014613), *gnd* (U00096, AE005174, AE006468, and AE014613), *gyrB* (U00096), *icdA* (U00096), *mdh* (U00096), *purA* (U00096) and *recA* (U00096). Some of the primers used to amplify these loci were obtained from the *E. coli* MLST website (http://mlst.ucc.ie/mlst/dbs/Ecoli) [48] while others were designed *de novo* to target other housekeeping genes (Table S1). The PrimerSelect program of the DNASTAR Lasergene 7 package (DNASTAR, Inc., Madison, WI) was used for primer design from previously sequenced *E. coli* and *S. enterica* genomes. PCR protocols to amplify *adk*, *gyrB*, *mdh*, *purA* and *recA* were performed according to the procedure specified by Wirth *et al.* [48] with minor modifications for annealing temperatures (Table S1). Thermal cycling conditions for the *aph*, *icdA* and *gnd* genes were as follows: an initial denaturation cycle at 94°C for 5 min, followed by 30 cycles at 94°C for 1 min, annealing temperature (according to each specific primer set Table S1) for 1 min and 72°C for 1 min with a final cycle of 72°C for 7 min. Platinum Taq DNA polymerase (Invitrogen, life technologies) was used to amplify all genes. PCR products were sequenced by High Throughput Genomics Unit at the University of Washington (http://www.htseq.org).

### Sequence Edition

The sequences were obtained in forward and reverse directions, and electrophenograms for all sequences were visually inspected for consistency between strands, with any ambiguous nucleotides being resolved by re-sequencing. The SeqMan program of the DNASTAR Lasergene 7 package (DNASTAR, Inc., Madison, WI) was used to edit, trim, and assemble each sequence. The ClustalW2 program of Bioedit software version 7.0.9.0 was used to align all the sequences. The coding sequences used for the housekeeping gene fragments were read in-frame.

### Accession Numbers

The GenBank IDs for the genes sequenced in this study are as follows: Accession numbers: *adk* (JQ606828, JX001409–JX001414), *aph* (JX001416–JX001422), *gnd* (JX001424–JX001430), *gyrB* (JX001432–JX001438), *mdh* (JX001440–JX001446), *purA* (JX001448–JX001454), *recA* (JX001456–JX001462), *icd* (JQ860228, JX001470–JX001474), and 16S rRNA (JX001463–JX001469).

### Phylogenetic Analyses

Phylogenies based on both individual and concatenated allele sequences (haplotype) were reconstructed using PhyML software, version 3.0 [49]. ModelTest [50] was used to select the optimal evolutionary model by evaluating the selected parameters using the Akaike Information Criteria (AIC). A corrected version of the AIC (AICc) was used for each locus sequence data set since the sample size (n) was small compared with the number of parameters (n/K<40). This approach suggested the following models: *adk* (TIM2ef+G), *aph* (TIM2+G), *gnd* (TIM3ef+I+G), *gyrB* (GTR+I+G), *icdA* (TIM2ef+I+G), *recA*, *purA* and *mdh* (TIM3+I+G), and the complete concatenated sequences data set (GTR+I+G). The robustness of nodes was assessed using 1000 bootstrap replicates for each locus separately and for the concatenated data set. This analysis was run using the same version of the PhyML software. Finally, trees were edited using MEGA version 5.01 [51].

In order to assess the role of recombination in the studied strains, the linkage model “STRUCTURE” [52] was run assuming a K value between 2 and 8 (20 replicates per value of K) populations with a burning of 25,000 iterations and 50,000 MCMC subsequent iterations. The optimal value was *K* = 4 as ascertained by comparing the posterior probabilities of the data given each value of *K* from 2 to 8 (Figure S1). Assignment of haplotypes to each ancestral population was performed as described by Wirth *et al.* [48].

For phylogenetic studies other enterobacteria sequences were also used. The sequences for the following strains were downloaded from the National Center of Biotechnology Information: *Citrobacter freundii* ballerup 7851, *C. freundii* 4_7_47CFAA, *C. freundii* GTC 09479, *C. freundii* GTC 09629, *C. freundii* MTCC1658, *C. koseri*, *C. rodentium*, *Citrobacter* sp. 30_2, *C. youngae*, *Cronobacter sakazakii*, *Escherichia alberti*, *E. fergusonii, E. coli* strains APEC O1, CFT073, B171, BL21-Gold(DE3), HS, O157:H7 strains, EDL933 and Sakai, K-12 substrains, MG1655, O111:H-, *Klebsiella pneumoniae* strains 342, NTUH-K2044, MGH 78578, *rhinoscleromatis, Shigella boydii, Sh. flexneri, Salmonella enterica* subsp. Arizona, *S. enterica* serovars: Gallinarum, Kentucky, Typhimurium LT2, Newport, Paratyphi A, Saintpaul, Schwarzengrund, Typhi strains CT18 and Ty2, Virchow and Weltevreden. Accession numbers can be found in Table 2.

**Table 2:** Accession numbers of the chromosome sequences used in this study.

Accession number	Organism and strain	Reference
CACD01000000	*Citrobacter freundii* ballerup 7851	[29]
ADLG01000000	*Citrobacter freundii* 4_7_47CFAA	GenBank
AOMS01000000	*Citrobacter freundii* GTC 09479	GenBank
AOUE01000000	*Citrobacter freundii* GTC 09629	GenBank
ANAV01000000	*Citrobacter freundii* MTCC1658	[79]
CP000822	*Citrobacter koseri* ATCC BAA-895	GenBank
FN543502	*Citrobacter rodentium* ICC168	[29]
ACDJ01000004	*Citrobacter sp*. 30_2	GenBank
ABWL02000006	*Citrobacter youngae*	GenBank
CP000783	*Cronobacter sakazakii*	[99]
ABWM02000041	*Enterobacter cancerogenus*	GenBank
CP000653	*Enterobacter sp*. 638	GenBank
ABKX01000005	*Escherichia albertii*	GenBank
CP000468	*Escherichia coli* APEC O1	[100]
AE014075	*Escherichia coli* CFT073	[101]
AAJX02000011	*Escherichia coli* B171	GenBank
CP001665	*Escherichia coli* BL21-Gold(DE3)	GenBank
AE005174	*Escherichia coli* O157:H7 EDL933	[44]
BA000007	*Escherichia coli* O157:H7 Sakai	[102]
CP000802	*Escherichia coli* HS	[103]
U00096	*Escherichia coli* str. K-12 MG1655	[43]
AP010960	*Escherichia coli* O111:H-	[104]
CU928158	*Escherichia fergusonii*	GenBank
CP000964	*Klebsiella pneumoniae* 342	[105]
AP006725	*Klebsiella pneumoniae* NTUH-K2044	[106]
CP000647	*Klebsiella pneumoniae* MGH 78578	GenBank
ACZD01000147	*Klebsiella pneumoniae* subsp. *rhinoscleromatis*	GenBank
CP000036	*Shigella boydii* Sb227	[107]
AE005674	*Shigella flexneri* 2a str. 301	[108]
CP000880	*Salmonella enterica* subsp. Arizonae	GenBank
AM933173	*Salmonella enterica* subsp. enterica serovar Gallinarum	[60]
ABEI01000005	*Salmonella enterica* subsp. enterica serovar Kentucky	[109]
AE006468	*Salmonella enterica* subsp. enterica serovar Typhimurium LT2	[41]
CP001113	*Salmonella enterica* subsp. enterica serovar Newport SL254	[109]
FM200053	*Salmonella enterica* subsp. enterica serovar Paratyphi A AKU_12601	[110]
ABAM02000001	*Salmonella enterica* subsp. enterica serovar Saintpaul SARA23	[109]
CP001127	*Salmonella enterica* subsp. enterica serovar Schwarzengrund CVM19633	[109]
AL513382	*Salmonella enterica* subsp. enterica serovar Typhi CT18	[46]
AE014613	*Salmonella enterica* subsp. enterica serovar Typhi Ty2	[45]
ABFH0200000	*Salmonella enterica* subsp. enterica serovar Virchow	[109]
CP001120	*Salmonella enterica* subsp. enterica serovar Heidelberg SL476	[109]
ABFF01000007	*Salmonella enterica* subsp. enterica serovar Weltevreden	[109]

### DNA Polymorphism Analyses

The number of substitutions per site in terms of both synonymous and nonsynonymous changes was calculated using DnaSP v 5.10 in non-overlapping windows of 30 bp [53].

## Results

The isolates used in this study came from a previous study in which the aim was to determine the phenotypic changes suffered by the atypical *C. freundii* FMU108327 isolate systematically (Supporting information). The experimental environment consisted of a serial transfer regime in which the *C. freundii* was subjected to repeated subsequent cultivation followed by the selection of single colonies (Figure 1). The detail can be found in the method section above. Considering the variability in phenotypes, the original atypical *C. freundii* FMU108327/P isolate was selected with five derivative isolates from two different branches (Figure 1).

### Biochemical Variability of Atypical FMU108327 Strain

The six selected isolates and two reference strains were streaked onto both blood agar and SS agar plates and incubated at 37°C. On inspection the following day, the original FMU108327/P (P) strain and its derived isolates, as well as the reference *Citrobacter freundii* E9750 and *Salmonella* Typhimurium LT2 strains produced unique and uniform colonies. Additionally, in SS agar, the strains of *C. freundii* E9750, FMU108327/P, and the isolates FMU108327/A_1_ and FMU108327/B_1_ yielded pink colonies indicating that these bacteria did ferment lactose. However, the FMU108327/A_10_, FMU108327/B_2_ and FMU108327/B_10_ isolates produced colourless colonies, indicating that these did not ferment lactose and/or colonies with a black dot indicative of iron sulphide production. Regarding reaction to KCN (potassium cyanide), the *C. freundii* strains and all derivative isolates grew, whereas *S.* Typhimurium did not. In terms of decarboxylation of lysine, which is the differential biochemical test between *Salmonella* spp. and *Citrobacter* spp., C. *freundii* E9750 and the isolates FMU108327/P and FMU108327/A_1_ were negative, while FMU108327/A_10_, FMU108327/B_2_, FMU108327/B_10_, and *S.* Typhimurium were positive. The FMU108327/B_1_ isolate decarboxylated lysine poorly resulting in a weak positive reaction. The β-galactosidase (ONPG) test helps to differentiate between *Salmonella* and *Citrobacter* cultures, particularly strains that ferment lactose slowly or fail to utilize it. The production of ONPG was positive for C. *freundii* E9750, FMU108327/P, FMU108327/A_1_ and FMU108327/B_1._ However, the isolates FMU108327/A_10_, FMU108327/B_2_, FMU108327/B_10_, and *S.* Typhimurium were all negative for ONPG production. These results confirm those obtained previously (Table1) (Supporting information).

### I-*Ceu*I Restriction Fragments of FMU108327/P and FMU108327/A_10_ Isolates

The *Ceu*I restriction endonuclease recognizes a 26-bp sequence from position 1911 to 1936 of the 23S rRNA gene in *rrn* operons, with the number of *Ceu*I fragments usually representing the number of *rrn* operons. The *rrn* skeleton of *S.* Typhimurium LT2 [37], [38] had been previously mapped using chromosome digestion with *Ceu*I endonuclease and PFGE. Data from this strain was used to construct *rrn* skeletons of two isolates: FMU108327/P and FMU108327A_10_. Initially, the *Ceu*I chromosomal profiles of all studied isolates were carried out but the results showed only 2 different profiles among the studied isolates, one of 7 bands and the other of 8 bands. Therefore, only 2 representative isolates of each profile were selected for further analyses.

The DNA digestion of *S.* Typhimurium LT2 yielded a profile with 7 bands; the molecular weight of each band was the same as that of *S.* Typhimurium LT2 reported by Liu (Figure 2a, 2b) [54]. *C. freundii* E9750 NCTC strain yielded 8 fragments, indicating that *C. freundii* contains 8 *rrn* operons in its chromosome. The isolate FMU108327/P also yielded 8 fragments while the derivative isolate FMU108327/A_10_ only yielded 7 fragments: A to G (Figure 2a). The calculated chromosome size was 4,739 kb for *C. freundii* E9750 and 4,572 kb for FMU108327/P, while the derivative isolate FMU108327/A_10_ had a chromosomal size of 4,520 kb (Figure 2b).

The corresponding individual fragments from the isolate FMU108327/P and the derivative isolate were determined by probing the Southern blotting membranes from PFGE that used PCR products of marker genes from *S.* Typhimurium LT2 (Table S1), since their location in the chromosome is known. The marker genes were: *metB* gene corresponding to band E, *metC* gene to band B, *metE* gene to band D, *metH* gene to band G, and *fliC* gene to band A (Figure 2).

The results obtained from these hybridizations indicated that *C. freundii* E9750 and FMU108327/P strains presented an extra cleavage that probably divides the 740 kb fragment (G) into smaller fragments, G (154 kb) and H (584 kb). Interestingly, the derivative isolate FMU108327/A_10_ did not show this additional cleavage site in fragment G, maintaining its size at 740 kb, and its *rrn* skeleton resembling that of *S. enterica* (Figure 2a, 2c).

### Chromosomal Arrangement of FMU108327/P and FMU108327/A_10_ Isolates

PCR was used to determine the order of *Ceu*I fragments and the *rrn* arrangements observed in these bacteria. To determine the chromosomal arrangement of the strains, genes adjacent to *rrn* operons were amplified (Table S1). In this way, the PCR assay confirmed the results obtained from hybridization on *Ceu*I PFGE and showed the arrangement of the FMU108327/P and FMU108327/A_10_ isolates.

Regarding isolate FMU108327/A_10_, the hybridization patterns and PCR gene assays were similar to those observed for *S. enterica* strains. The data also indicated that the order of the *rrn* operons in this strain was BCDEFG; the positions of fragments B and G were adjacent to fragment A (Figure 2a) to form a circular chromosome. Meanwhile, atypical FMU108327/P showed some differences. Although the majority of the selected genes hybridized according to expected fragments (Table S1), there were a couple of differences: *yrdA* failed to hybridize in fragment *Ceu*I-C, *mobB* did not hybridize in fragment E nor in any other fragment of the profile, although PCR assays on total genomic DNA indicated that the genes were present in the FMU108327/P isolate. These differing hybridization patterns indicate chromosomal rearrangements within the isolates concerned.

### Presence of the Adaptative Genes *cadA* and *lacY*

Lactose fermentation, decarboxylation of lysine, and the utilization of potassium cyanide (KCN) are the most important criteria for differentiating *Salmonella* spp. (**−**, +, **−** respectively) from *C. freundii* (+/−, **−**,+respectively) (Table 1). Due to the atypical isolates in this study exhibiting changes in some of these biochemical phenotypes, the presence of the *cadA* gene that encodes lysine decarboxylase and the *lacY* gene that encodes lactose permease was investigated using PCR. The results showed the presence of *cadA* in FMU108327/P and in all of its derivatives, as well as in *S*. Typhimurium LT2, while the *C. freundii* strain E9750 was negative. With regards to the *lacY* gene, *C. freundii* E9750, FMU108327/P and its derivatives were positive while *S*. Typhimurium LT2 was negative (Figure S2).

### Phylogenetic Reconstruction and Mutation Distribution

**Figure 3:**
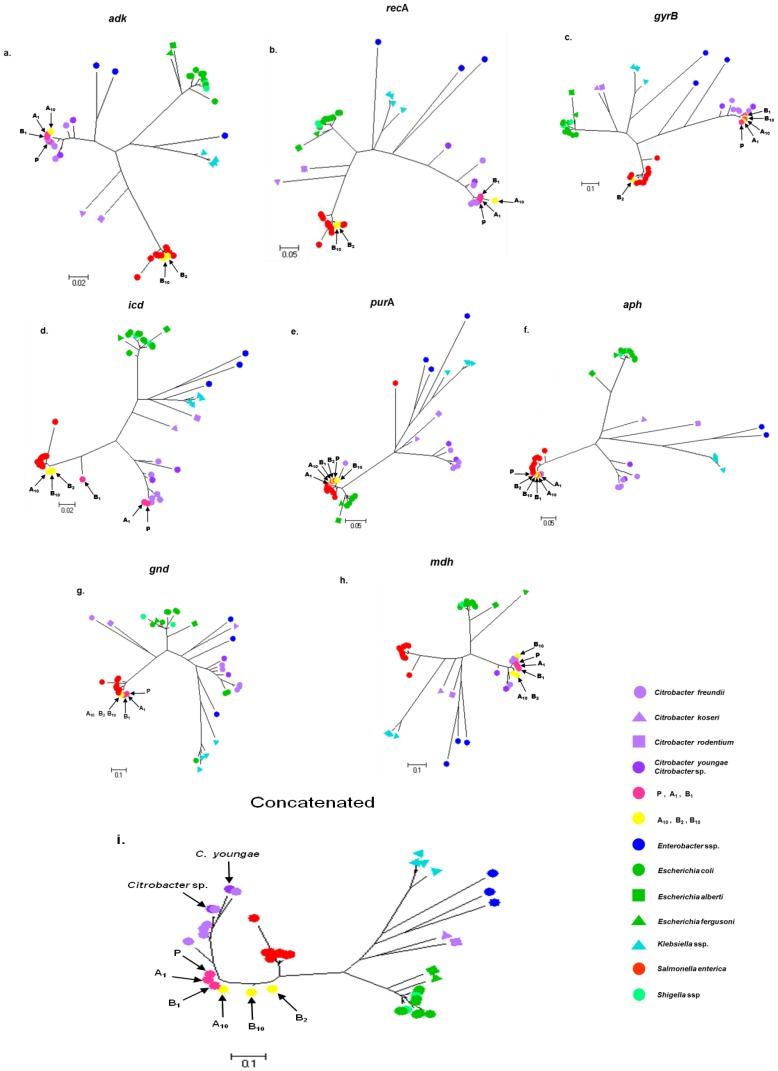
Individual and concatenated maximum likelihood trees. All of the trees were constructed in Mega 5.01 for a) *adk*, b) *recA*, c) *gyrB*, d) *icd*, e) *purA*, f) *aph*, g) *gnd*, h) *mdh* and i) concatenated genes. The assigned names of the genus in which these alleles occur are shown. The scale bar represents the number of substitutions per site.

The phylogenetic analysis of the isolates showed different relationships between isolates depending on the sequenced genes. For example, when *adk* and *recA* genes were analysed (Figures 3a, 3b), the FMU108327/B_2_ and FMU108327/B_10_ isolates grouped in the *Salmonella* clade, while the FMU108327/P, FMU108327/A_1_, FMU108327/A_10_, FMU108327/B_1_ isolates and *C. freundii* E9750 clustered in the *Citrobacter* clade. For *gyrB* (Figure 3c), all of the isolates were located in the *Citrobacter* clade except for the FMU108327/B_2_ isolate. In the case of the *icd* gene (Figure 3d), FMU108327/B_2_, FMU108327/B_10_ and FMU108327/A_10_ isolates grouped in the *Salmonella* clade. Considering the *purA*, *aph* and *gnd* genes (Figure 3e–g), all the isolates (FMU108327/A_1_, FMU108327/A_10_, FMU108327/B_1_, FMU108327/B_2_ and FMU108327/B_10_) including the parental strain (FMU108327/P) grouped more closely to *Salmonella*, as did *C. freundii* E9750 when considering the *purA* and *aph* genes. Finally, with regard to the *mdh* gene (Figure 3h), all of the studied isolates (including the reference species *C. freundii*, *C. youngae* and *Citrobacter* spp.) formed a single cluster that diverged away from the *Salmonella* group.

Although there were alleles that could be clearly assigned to one genus, in the phylogenetic reconstruction using the concatenated sequences, the haplotypes did occupy intermediate positions in the PhyML tree from the *Citrobacter* spp. and the progenitor strain (FMU108327/P) clade to the *Salmonella* spp. clade (Figure 3i). This observation of intermediate clustering in the tree of the concatenated genes is also congruent with the phenotypic variation that the derivative isolates presented.

Amazingly, the 16S rRNA sequences showed the same “undefined” nature that was observed in the tree of concatenated housekeeping genes as they did not form a clear sequence cluster [55]. The FMU108327/A_10_, FMU108327/B_2_ and FMU108327/B_10_ isolates (haplotypes closer to *Salmonella*) showed 99% similarity to *S.* Typhimurium LT2 (AE006468) [41] and 98% similarity to both *C. koseri* (CP000822) and *C. rodentium* (FN543502) [29]. When the sequence of the FMU108327/P isolate was compared with its derivative isolates, FMU108327/A_10_, FMU108327/B_2_ and FMU108327/B_10_, similarity was reduced to 97.6%. Meanwhile, the similarity among the FMU108327/P, FMU108327/A_1_ and FMU108327/B_1_ isolates (haplotypes closer to *Citrobacter)* and the *C. freundii* MRB0903 strain (GU126681) was 99.8%. Finally, the sequence similarity between the *C. freundii* MRB0903 strain and the FMU108327/B_10_ isolate was 97.4%.

**Figure 4:**
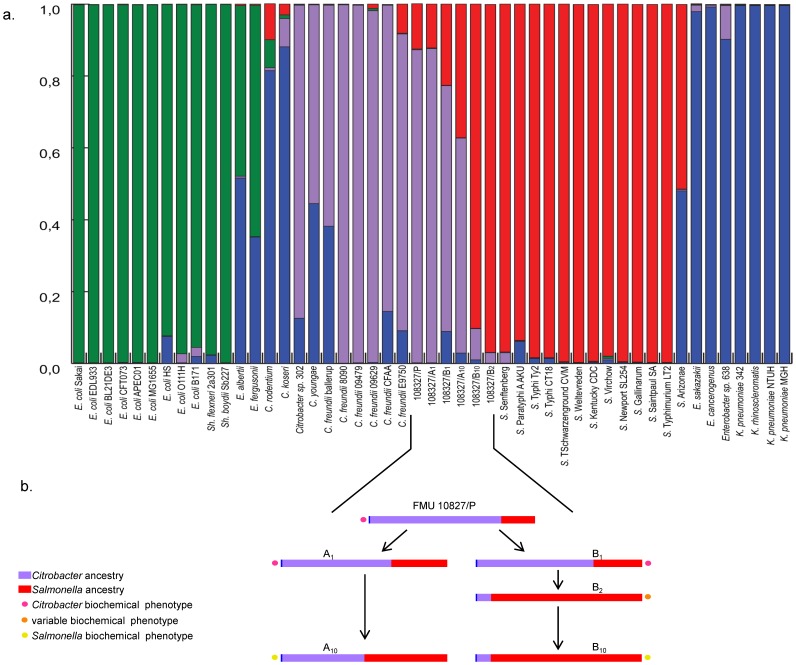
Proportion of ancestry inferred by STRUCTURE. Results of the STRUCTURE analysis assuming 4 populations, a) every vertical line represents each of the 49 isolates and indicates the proportion of ancestry from the 4 ancestral populations, using the following colors: green for *E. coli*, blue for *Enterobacter* spp. and *Klebsiella* spp., purple for *Citrobacter* spp. and red for *Salmonella* spp.; b) It shows the proportion from *Citrobacter* and *Salmonella* ancestries in our isolates, as well as their biochemical phenotypes. It is interesting to highlight the A10 and B2 isolates. In spite of the fact that A10 derivative isolate has 60% identity with *Citrobacter* ssp., it displayed Salmonella's biochemical phenotype which has remained stable until now; in addition we have the B2 derivative isolate which had a genotype with a majority of identity to *Salmonella* ssp genus and its biochemical phenotype was variable, thus preventing establishing some biochemical identity.

In order to understand the role of genomic flux in the dynamics of the studied isolates, a more formal species assignment was performed using the linkage model of Bayesian clustering algorithm STRUCTURE (Figure 4) [52]. The 6 isolates in the study formed two groups. In the first group, *C. freundii* E9750 (83%) and P (87%) strains and the A_1_ (87%) and B_1_ (69%) isolates had more than 60% identity with the *Citrobacter* genus while the remaining proportion identified with *Salmonella*. *C. freundii* CFAA strain was also included in this group and had 85% identity with *Citrobacter* and 15% with *Enterobacter/Klebsiella*. The second group that included the B_2_ and B_10_ isolates presented 90% identity with the *Salmonella* genus. It is important to highlight that the A_10_ derivative isolate showed 60% identity with *Citrobacter* ssp., 37% with *Salmonella* ssp and the remaining 3% with *Enterobacter/Klebsiella* group. In addition, *C. freundii* ballerup also showed this hybrid nature, this strain presented 62% identity with *Citrobacter* genus and the remaining 32% with *Enterobacter* group (Figure 4).

**Figure 5:**
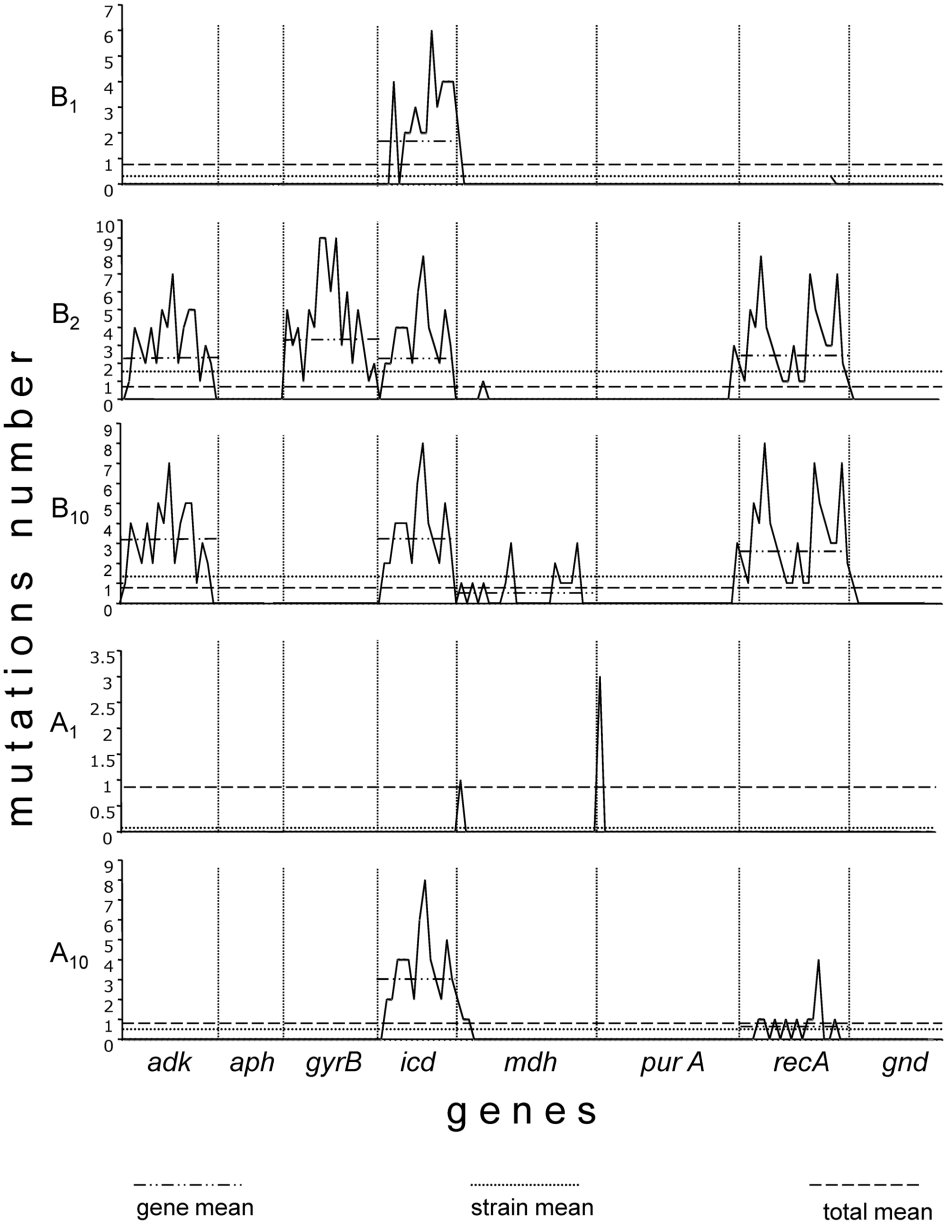
Mutation frequencies. Mutation frequencies for each of the 30 positions in 8 genes of 5 strains are shown. The isolates with the greatest number of mutations were B_2_ and B_10,_ which presented the most diversified genotype, and the genes with the greatest number of mutations were *adk*, *icd* and *recA*.

The nested distribution analysis of the point mutations by region (30 pb sliding window) per gene, between genes and between isolates showed differences at all three levels (Figure 5). The isolates that accumulated more point mutations were FMU108327/B_2_ and FMU108327/B_10_, while the FMU108327/A_10_ isolate accumulated few mutations; in only 2 markers. The molecular marker that accumulated the most mutations of all isolates was the *icd* gene. The frequency of synonymous against nonsynonymous mutations also differed considerably, with more synonymous mutations being found in all genes and all isolates, suggesting that at the molecular level, most of the variation was neutral (Table S2).

## Discussion

Phenotypic variation of many phenotypic markers is a major adaptive strategy of pathogenic and commensal bacteria [2], [3]. It has been shown that such variants are produced at high frequencies and in a reversible manner by bistability, bet-hedging, phase variation, large accumulation of mutations or hyper-variable methylation in specific regions of the genome [3], [56].

In the phenotypic diversification study (supporting information), atypical *C. freundii* FMU108327/P was shown to undergo frequent and reversible phenotypic changes (colonial morphology, biochemical characteristics) from the parental strain to its derivatives and vice-versa. This on-off molecular mechanism suggests that the changes in phenotypic expression are induced by a fluctuating environment, but surviving under these conditions poses challenges. Hypothetically, a bacterial population under such challenging conditions could produce off-spring with variable phenotypes to ensure that at least one of them, with a new phenotype, will be able to adapt to the new environment and become stable [57], [58]. This has been shown previously in some bacterial species, such as *C. jejuni* [59] and *Salmonella enterica* [58], [60] or more recently in *Citrobacter rodentium* [30] in which gene flux has been reported due to IS elements and prophage/phages, which are able to insert randomly throughout the chromosome and plasmids. The reshuffle of genes in both species resulted in the significant loss of functional genes associated with virulence and metabolic functions [29], [30].

Many questions about genetic, evolutionary, and ecological mechanisms behind phenotypic variation remain unresolved but it is possible that variable phenotypes aid in the survival of the bacteria under adverse conditions or, in pathogenic microorganisms might help them to increase their virulence or evade the immune responses of their hosts. In addition, these variations may serve to balance the benefits and disadvantages of a certain phenotype and in the case of competition, there may be a trade-off between the benefits of the generation of phenotypic variation and genetic diversity [3], [57].

There are four bacterial mechanisms that can shape the formation of new genotypes: a) chromosomal rearrangements; b) point mutations; c) homologous and non-homologous recombination; and d) horizontal gene transfer [61].

Concerning the first mechanism, chromosomal rearrangements are common in enterobacteria [62]–[67]. Frequently, strict host-specific serovars of *S. enterica,* such as serovars Typhi, Pullorum, and Gallinarum, have large-scale genomic rearrangements due to recombination between rRNA (*rrn*) operons [68]–[71], while generalist serovars from animal and natural sources worldwide have stable genomes at the *rrn* level [72]. Interestingly, similar rearrangements in *rrn* operons have been found in other species, such as *E. coli* and *V. cholerae* among others [73]–[78].

In the current study, *Ceu*I fragment analysis revealed that chromosome sizes and the structure of the included isolates were similar. Nevertheless, the extent of rearrangement in *rrn* regions was unexpected. The chromosome of *C. freundii* FMU108327/P was found to have eight *Ceu*I fragments showing a very similar *rrn* profile to that presented by *C. freundii* NCTC E9750 (Figure 2). These data agree with that reported by other authors [31]. Contrary to what had been expected, the chromosome of the derivative isolate FMU108327/A_10_ was found to have seven *rrn* operons, and appeared to have lost a *Ceu*I site resulting in a similar profile to that observed in *S. enterica serovar* Typhimurium [72]. This genetic event could have resulted from the deletion of a segment due to recombination between two *rrn* operons in the same replichore, thus forming a circular fragment, followed by reinsertion of a deleted segment into another location.

The second bacterial mechanism to form new genotypes, namely point mutation, can cause the loss of endonuclease cleavage sites. In the current study, this mechanism of genotype formation cannot be discarded until full genome sequences can be conducted to determine which bases might have changed in the FMU108327/P isolate and in its derivative FMU108327/A_10_ that led to one of the *Ceu*I sites no longer being cleavable.

Despite the existence of a hypothesis concerning the order and structure of the chromosome being subject to selective pressure, in enterobacteria it is common to find variation in the distribution of ribosomal operons (*rm*). *C. freundii* is not an exception to these kinds of rearrangements, as shown in the above results and in the data from Kumar *et al.* [79] regarding the sequence of *C. freundii* MTCC 1658 that had 10 rRNA operons or in the results from Bai *et al.* [31] that found in *C. freundii* CF72 and CF74 8 rRNA operons. Previous studies have shown that genomic changes do occur in natural bacterial populations [80], [81]. However, the majority of these are deleterious and as a result, only a very small proportion of the bacterial population undergoing genomic changes may survive under specific natural selection pressure [80], [81].

One interesting question that remains unanswered is what triggers such changes in the chromosomal synteny and what is its biological importance. The conclusions from the current study suggest that the bacteria were placed under thermal stress, which acted as a selective pressure, leading to the selection of some mutations that enhanced survival in such a specific environment, which in turn led to genomic diversification and special genomic structure that adapted better to the local environmental setting [80]. Recently, Ciu *et al.* [82] showed the biological importance of this type of genomic rearrangement in *S. aureus* strains. They demonstrated that a large-scale inversion of the chromosome triggers the bacterial phenotype “on-off switch”, including colony morphology, antibiotic susceptibility, and the expression of dozens of genes.

On analysing the sequences of the eight housekeeping genes from the isolates, an astounding genetic diversity was found from a single clone. Moreover, the majority of the point mutations were synonymous, meaning that most of them did not change the enzyme function. This provided clear evidence of selective constraint on amino acid replacements. However, some of these mutated genes produced diversity in genotypes, some of which are closer (but not equal) to the genotypes of the *Salmonella* genus. This large quantity of mutations could only be explained by a hyper-mutant genotype induced and maintained by repair systems, such as SOS [83]–[85] and the DNA mismatch repair system (MMR) [86]. The MMR system is well known for its role in maintaining genetic stability, but defects in this system lead to high mutability. Bacteria having defects in MMR are mutators or hypermutator cells, which have elevated rates of spontaneous mutation and recombination enabling a diversity of mobile DNA to be incorporated into the genome. The MMR system in particular has been widely studied in *Salmonella* Typhimurium [86]–[89]. The results show that the conversion of *mutL* gene between functional and defective alleles through the deletion of one of the three tandem 6 bp repeats within the gene sequence (via slipped-strand mispairing) may act as a genetic switch between genetic stability and a mutability state [87]–[89]. However, a hyper-mutation state may be temporary, leading to genomic diversification. The repetitive structure within the sequence of *mutL* enables it to function as a genetic “on-off” switch at a population level by the selection of existing cells possessing the favorable traits, which may allow return to the original wild-type phenotype [88], [89]. Additionally, in *Salmonella* Typhimurium, a hyper-mutability state, as well as prophage/phage flux plays an important role in offspring diversity. Such a state is also related to nutritional function loss, as well as shifts to alternative biochemical pathways and colony morphology diversity [80], [89].

All of the genes involved in SOS responses (*recA*, *lexA*) and the MMR system (*mutS*, *mutL*, *mutH*) are found in the genomes of *Citrobacter* species that have been sequenced to date. The information of corresponding genes was compiled from the respective published genome sequences. These data are relevant, since the presence of such systems in *C. freundii* may explain the rapid and large number of accumulated mutations, as well as diversification within just a few generations.

Diversity in the16S *rrn* gene within a species has already been observed [90]–[95]. In *V. vulnificus* [91], [92], this heterogeneity can be related to virulence, while in *Bacillus* [93] the existence of some specific nucleotides in the 16S rRNA gene is related to the capacity to grow at both low and high temperatures. In *Aeromonas* spp. [94], a range of diversity between 96.7% and 100% has been reported, while in clinical strains of *S. anginosus* [95], micro-heterogeneity has been related to the phenotypic characteristics and the adaptation of the organism to different niches. Unfortunately, in the current study, it was not possible to relate the observed micro-heterogeneity in the sequences of 16S *rrn* of *C. freundii* and its derivative isolates to any phenotypic characteristic or function.

The phylogenetic analysis of *C. freundii* showed that although this species was grouped in one cluster, it seemed to be more diverse and dynamic than the *Salmonella* and *E. coli* clades. In fact in this clade, *C. youngae* and *Citrobacter sp*. clustered too, but *C. rodentium* and C. *koseri* did not and were more distantly related. Therefore, these data indicate that *Citrobacter* is a polyphyletic genus. In general, these results were consistent with those previously reported [29] with some differences in the clonal relationship between *C. freundii* and *Salmonella sp*., probably due to the analysis including more *C. freundii* strains in the current study. In addition, the results from the analyses of Maximum Likelihood of the FMU108327/P and its derivative isolates reveal the hybrid nature of these genotypes (Figure 3). Moreover, in the concatenated tree (Figure 3i); even though the haplotypes clustered within the *C. freundii* branch, they were located in an intermediate gradient between the *Citrobacter* and *Salmonella* clades. Finally, and in order to verify the presence of hybrid genotypes among the isolates, these sequences were analysed using the linkage model of Bayesian clustering algorithm “STRUCTURE”. The “STRUCTURE” (Figure 4) analysis also supported the existence of this hybrid group. In general, moving towards the interior of the *Citrobacter* group, each strain was very heterogeneous, showing that some of their alleles were imported from other species [96]. Thus, this finding could explain the hybrid nature of this genetic pool as a result of convergence with other enterobacteria, a phenomenon that seems to be common in enteric bacteria, which are seen to be dynamic entities [97]. For example, in *C. rodentium, E. coli* or *S. typhi,* and *S. paratyphi*, this phenomenon has been demonstrated in terms of their process of pathogenesis due to the high frequency of genetic interchange, although in all cases an immediate common ancestry does not exist [29], [98].

In studies that had analysed phenotypic variation mechanisms, it has been shown that certain bacterial species can shape their phenotype in two or more stable states depending on the environment [2], [4]. The results from the current study opens the discussion further and questions if the phenotypic variation mechanisms, chromosomal rearrangements, large accumulation of mutations and genomic flux may enable a bacterial population to overcome the barriers that exists at the species level and generate new genotypes that, due to convergence, are closer to other species. Alternatively, it may be that atypical *C. freundii* strains exhibit different genotypic varieties of the same species co-existing in a stable way in the same time frame but expressing different phenotypes that are dependent on external environmental factors in which they are living.

Further work is needed to validate some of the hypotheses outlined in this paper. It is necessary to demonstrate the dynamics that the bacteria follow in the diversification study in order to analyse population genetics in a representative sample of the isolates that were obtained in the phenotypic study.

In addition, the genome sequence and a genomic and transcriptomics approximation of some of the lineages from the phenotypic variation study should be carried out. Furthermore, more atypical *C. freundii* strains should be included in order to determine if these characteristics are specific to the strain or if it is a wider-ranging genus. This would be important for providing details of genetic events to improve understanding of the dynamics and mechanisms that shape the structure of genomes, the processes related to divergence, convergence and diversification, their biological importance, and in particular, in bacterial evolution and species boundaries.

Another important consideration is the clinical implications of isolate *C. freundii* strains with these genetic rearrangements and genomic flux. Is it possible that this genetic plasticity induces the pathogenic behavior of these atypical strains?

Until now, definitive classification of *C. freundii* has been difficult creating a taxonomic problem. Published data, together with the results from this current study, highlight the great diversity and plasticity of this species and offer possibilities for separating *C. freundii* as a species in a more precise way.

## Supporting Information

Figure S1 **Posterior probability of the number of genetic pools inferred by“STRUCTURE”.** The optimal value was *K* = 4 by comparing the posterior probabilities of the data given each value of *K* from 2 to 8.
(TIFF)

Figure S2 **PCR products of the adaptive genes** ***cadA*** **y** ***lacY*** **.** a) PCR products of *cad*A gene. Lanes: 1. *S*. Typhimurium LT2, 2. *C. freundii* E9750 NCTC, 3. FMU108327/A_1_, 4. FMU108327/A_10,_ 5. DNA ladder 100 bp, 6. FMU108327/B_1_, 7. FMU108327/B_2_, 8. FMU108327/B_10_, 9. *C. freundii* FMU108327/P. b) PCR products of *lac*Y gene. Lanes: 1 and 9. DNA ladder 100 bp, 2. *C. freundii* FMU108327/P, 3. FMU108327/A_1_, 4. FMU108327/A_10_, 5. FMU108327/B_1_, 6. FMU108327/B_2,_ 7. FMU108327/B_10,_ 8. C. *freundii* E9750 NCTC and 10. *S*. Typhimurium LT2.
(TIFF)

Table S1 **Primer sequences used in this study.**
(DOC)

Table S2 **Synonymous and Nonsynonymous substitutions.**
(DOC)

Text S1 (DOC)
