# Supplementary figures and images for: Genetic Characterization of Atypical Citrobacter freundii
Source: PLoS One. 2013 Sep 12;8(9):e74120. doi: 10.1371/journal.pone.0074120 (PMC3771896; doi:10.1371/journal.pone.0074120)

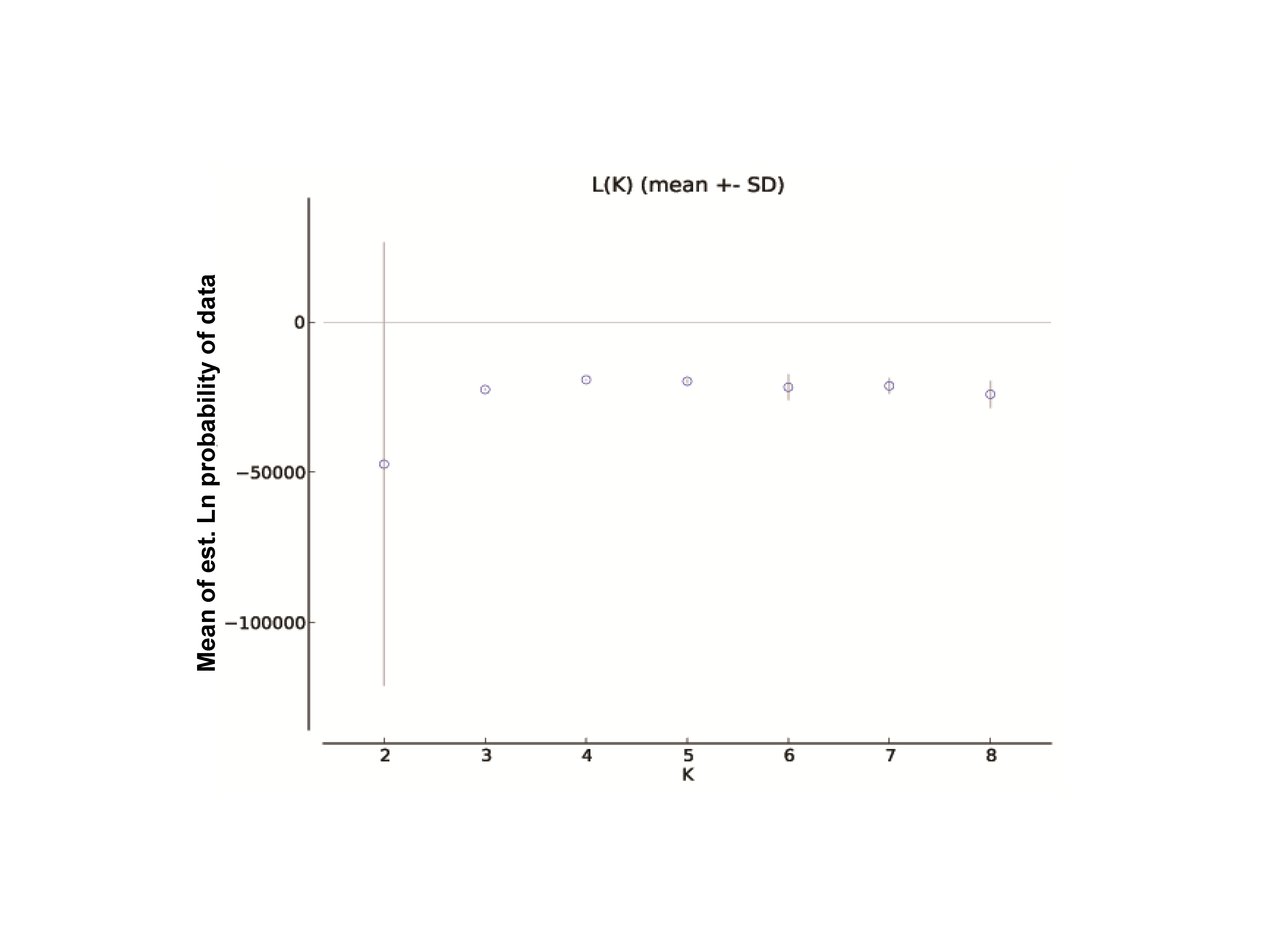

Supplement: Figure S1 — Posterior probability of the number of genetic pools inferred by“STRUCTURE”. The optimal value was K = 4 by comparing the posterior probabilities of the data given each value of K from 2 to 8. (TIFF) [file pone.0074120.s001.tiff]

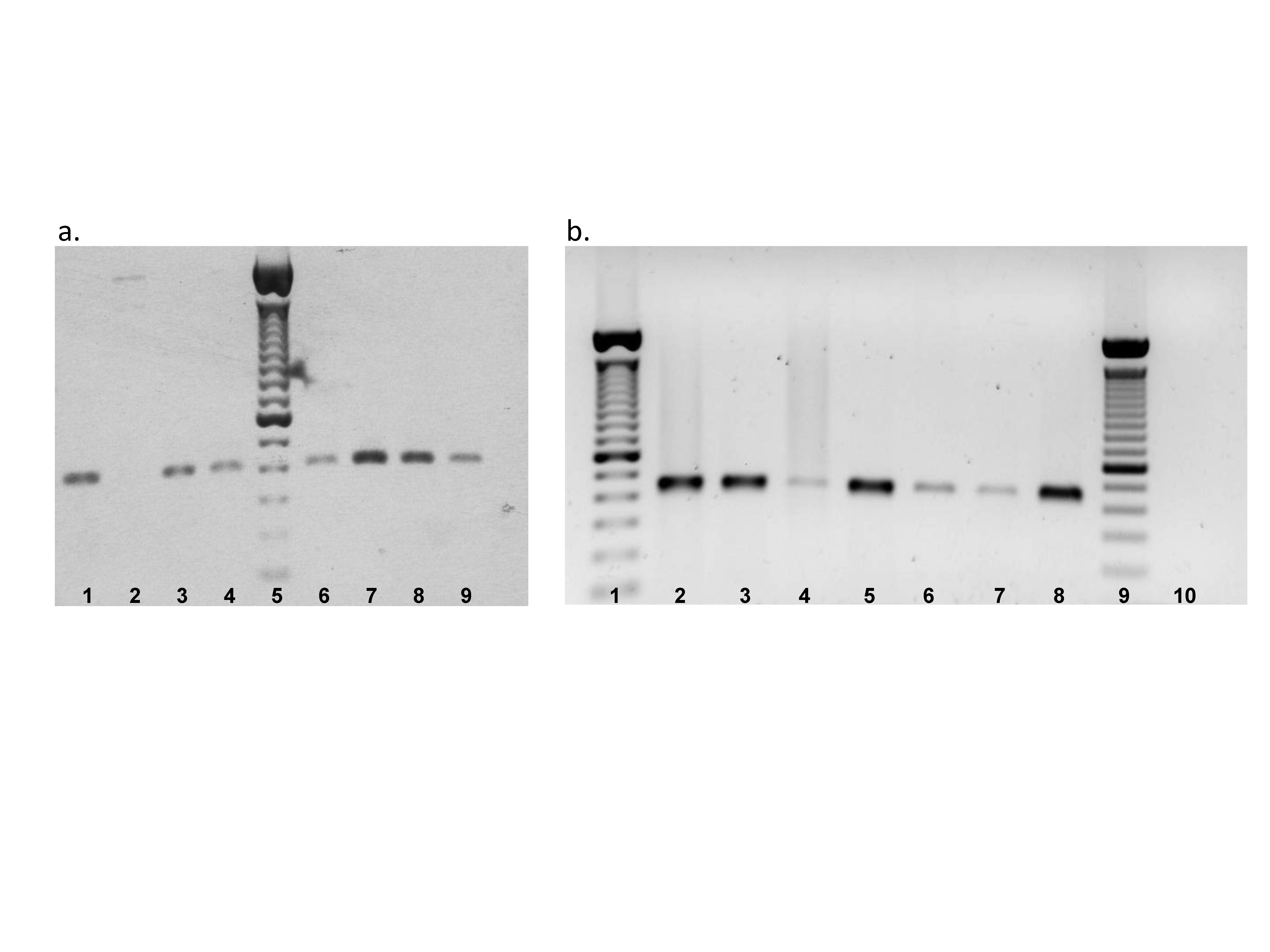

Supplement: Figure S2 — PCR products of the adaptive genes cadA y lacY . a) PCR products of cadA gene. Lanes: 1. S. Typhimurium LT2, 2. C. freundii E9750 NCTC, 3. FMU108327/A1, 4. FMU108327/A10, 5. DNA ladder 100 bp, 6. FMU108327/B1, 7. FMU108327/B2, 8. FMU108327/B10, 9. C. freundii FMU108327/P. b) PCR products of lacY gene. Lanes: 1 and 9. DNA ladder 100 bp, 2. C. freundii FMU108327/P, 3. FMU108327/A1, 4. FMU108327/A10, 5. FMU108327/B1, 6. FMU108327/B2, 7. FMU108327/B10, 8. C. freundii E9750 NCTC and 10. S. Typhimurium LT2. (TIFF) [file pone.0074120.s002.tiff]
