# Supplementary material for: Genetic Characterization of Atypical Citrobacter freundii
Source: PLoS One. 2013 Sep 12;8(9):e74120. doi: 10.1371/journal.pone.0074120 (PMC3771896; doi:10.1371/journal.pone.0074120)
Supplement: Table S1 — Primer sequences used in this study. (DOC) [file pone.0074120.s003.doc]

**Table S1.** Primer sequences used in this study.

| Gene | Forward primer sequence (5' - 3') | Reverse primer sequence (5' - 3') | Tm |  | Reference |
| --- | --- | --- | --- | --- | --- |
| **Marker genes** |  |  |  | **Location** |  |
| *fliC* | AATACCAACAGCCTCTCGCT | AGAGACAGAACCTGCTGC | 53.0°C | Ceu A | [40] |
| *metC* | CATGATGTTCCGGGGATTGTT | GCCCGCTGCTACCTGTAAAGTCTC | 59.3°C | Ceu B | This study |
| *metE* | GCGGCGGCAGCGGAAATGA | CAGGCAGCGCAGTAATCGTGTC | 62.4**°**C | Ceu D | This study |
| *metB* | GGGCGGCGCGTTTGACAGC | GTTTGCAGCCCGGAAGCCATTTT | 61.6**°**C | Ceu E | This study |
| *metH* | CGGCGGCGCGACCACTTC | TTTCTCGGCGCTCAATTTATCCAG | 61.6**°**C | Ceu G | This study |
| *rfb* | ATTGTGGCTGCAGGGATCAAAGAAATC | TAGTCRCGCTGNGCCTGRATYARGTTMGC | 60.0**°**C | Ceu A | [14] |
| **Oligonucleotide primers according to their genomic location relative to *rrn* operons** | | |  | **Location** |  |
| *hemG* | GCGCGAAATTGCCTCTTAT | GTTTATTTTGCCGACGATTTGT | 56.4**°**C | 5´*rrn*A | This study |
| *mobB* | ACCGCTACTCGCCATTG | AACGTGCTTTCTCTAACCA | 60.7**°**C | *3´rrn*A | This study |
| *murI/murB* | GCGTCGGTGGATTGTCGGTCTATGA | CCAGGCGCTCAGTAGTTGTTGTTCG | 61.0**°**C | 5´*rrn*B/3´*rrn*B | [64] |
| *yieP/yifA* | GCTCCAGGCTAATACGCATCACCAG | GCTGTTAGGGCACTTCACTTTGGCG | 60.4**°**C | 5´*rrn*C/3´*rrn*C | [64] |
| *yrdA* | CGATGTGAATTACGTTGCGATAGG | CTGTTTTACCGGACTGCCAAGATA | 60.0**°**C | 5´*rrn*D | This study |
| *acrF* | CTGGCGATAGGGCTGTTGGTTGAC | CACGATGCCGCTTACGCTGTTAGT | 58.7**°**C | 3´*rrn*D | This study |
| *purH* | CGGCGGCTTCTTTGCTTTCA | GTCCGCCGCGCTCTGCTC | 60.7**°**C | 5´*rrn*E | This study |
| *metA* | CAGGAAATTCGCCCGCTAAAGGTA | GTGGCGCCAGAAACGAATCATCAA | 58.6**°**C | 3´*rrn*E | This study |
| *clpB* | TTTGTTCTGGCGGCGCTTGAGTCT | GATATTTCGCCCCCGCCACCAG | 60.9°C | 5´*rrn*G | This study |
| *kgtP* | AGTTGCGCTTGAAGGTCGTAA | ACACAGCGCAGCCGTCATAAT | 57.3°C | 3´*rrn*G | This study |
| *yaeD* | GGGCTATGCGTTGGTGGTGGTGA | GTCCGCCGCGTTTTCTGCTTCT | 60.8°C | 5´*rrn*H | This study |
| yafB | CGCGGCGCTACAAACTGAG | CGGCGGTGGCGATAATGTC | 59°C | 3´*rrn*H | This study |
| **Housekeeping genes** | |  |  |  |  |
| *adk* | ATTCTGCTTGGCGCTCCGGG | CCGTCAACTTTCGCGTATTT | 66.5°C |  | [48] |
| *aph* | CTATTCCCCGGACAGCGACGATTAT | TGCGGCAGCGGTTTGTAGGTG | 61°C |  | This study |
| *gnd* | CGATGGCGCGGGTCACTAC | ATACAGCCCGCACGGAAAATCTT | 58.6°C |  | This study |
| *gyrB* | TCGGCGACACGGATGACGGC | ATCAGGCCTTCACGCGCATC | 64°C |  | [48] |
| *icd*A | GCGAAGAGATGGGCGTGAAGAAAAT | CAATACCGATACCGCCAACCTGAGC | 57.8°C |  | This study |
| *mdh* | ATGAAAGTCGCAGTCCTCGGCGCTGCTGGCGG | TTAACGAACTCCTGCCCCAGAGCGATATCTTTCTT | 66.5°C |  | [48] |
| *purA* | CGCGCTGATGAAAGAGATGA | CATACGGTAAGCCACGCAGA | 66.5°C |  | [48] |
| *recA* | CGCATTCGCTTTACCCTGACC | TCGTCGAAATCTACGGACCGGA | 58°C |  | [48] |
| *16S* | AGAGTTTGATCMTGGCTCAG | TACGGYTACCTTGTTACGACTT | 60°C |  | [113] |
| **Adaptive genes** |  |  |  |  |  |
| *cad*A | CCCCGTCGGCAGCATCTTTTAT | CCAGGTGGCGTTAGGCGTCTCTTT | 59.6°C |  | This study |
| *lac*Y | AGCCAGCCGCCGAAGCAACT | ACAATCAGCGGCGCAAAGAACAT | 58.6°C |  | [47] |
