# Supplementary material for: Genetic Characterization of Atypical Citrobacter freundii
Source: PLoS One. 2013 Sep 12;8(9):e74120. doi: 10.1371/journal.pone.0074120 (PMC3771896; doi:10.1371/journal.pone.0074120)
Supplement: Table S2 — Synonymous and Nonsynonymous substitutions. (DOC) [file pone.0074120.s004.doc]

**Table S2.** Synonymous and Nonsynonymous substitutions

| **Genes** | **Synonymous substitutions** | **Synonymous sites*** | **Nonsynonymous substitutions** | **Nonsynonymous Sites*** |
| --- | --- | --- | --- | --- |
| *adk* | 52 | 127 | 4 | 394 |
| *aph* | 1 | 90 | 0 | 314 |
| *gnd* | 0 | 109 | 0 | 326 |
| *gyrB* | 67 | 137 | 15 | 418 |
| *mdh* | 9 | 151 | 4 | 413 |
| *pur*A | 0 | 190 | 0 | 521 |
| *icd* | 46 | 96 | 4 | 309 |
| *rec*A | 72 | 161 | 0 | 496 |

*Total substitutions sites
