# Supplementary material for: Genetic Characterization of Atypical Citrobacter freundii
Source: PLoS One. 2013 Sep 12;8(9):e74120. doi: 10.1371/journal.pone.0074120 (PMC3771896; doi:10.1371/journal.pone.0074120)
Supplement: Text S1 — (DOC) [file pone.0074120.s005.doc]

**Supporting information**

Origin of the isolates of atypical *Citrobacter freundii*

In 1985, Cravioto *et al.* carried out a longitudinal study looking at the agents responsible for acute diarrhea in a cohort of 75 children. The study was carried out in a rural village in the state of Morelos, approximately 180 km southwest of Mexico City [32, 33]. A strain of *C. freundii* was isolated from this study, which exhibited atypical biochemical characteristics that impeded definitive bacterial identification leading to labelling these isolates as atypical *C. freundii*. All of the strains isolated from this study were labelled and stored on a sealed Dorset egg slant, in a temperature-controlled room.

Four years later, a study was started looking at the pathogenicity of *Citrobacter* ssp*.* [111], the objective of which was to determine if the atypical *C. freundii* strains were also associated with diarrhoeal processes. Five of the strains isolated during the Cravioto *et al*. study [32, 33] were used and these were identified as atypical *C. freundii* according to the changes seen in some of the differential biochemical tests such as lactose fermentation, decarboxylation of lysine, or the ability to grow in the presence of potassium cyanide (KCN). For this study, the selected strains were streaked onto McConkey and Salmonella-Shigella (SS) agar plates. Unique and perfectly isolated colonies were selected from each plate and inoculated into TSA tubes, Kligler Iron Agar (KIA) and peptone water. A 50µl aliquot was taken from the peptone water and inoculated into tetrathionate broth and incubated for 18 h at 37°C. Following incubation, the resulting isolates were selected and streaked onto McConkey and SS agar to cultivate unique colonies once again. From these plates, strains that presented different colony morphology were selected and inoculated into TSA tubes together with the biochemical tests: Kligler Iron Agar (KIA), fermentation of dulcitol (Dul) and sucrose (Suc), phenyl alanyl deaminase (PA), indole, H_2_S, urease (U), lysine decarboxilase (LCD), and β-galactosidase (ONPG). This process was repeated six times. The results from this study showed changes in the biochemical phenotype in some of the isolates; particularly in the biochemical markers of decarboxylated lysine and lactose fermentation [111].

Phenotypic variation study

Based on their previous results, Vázquez and Cravioto once again selected one of these atypical *C. freundii* isolates to determine in a systematic way the patterns and dynamics of the biochemical changes suffered by the atypical *C. freundii*.

The experimental environment of the phenotypic variation study consisted of a serial transfer regime in which the atypical *C. freundii* isolate was grown overnight in a sterile media of 3% skimmed-milk at 42°C. In order to have isolated colonies, the resulting culture was streaked onto SS agar plates and incubated overnight at 37°C and for a further 18 h at room temperature. Following incubation, all of the single colonies showing morphologic differences such as size, texture and consistency were selected (always inspected, selected and picked with the aid of a stereoscopic microscope in a sterile area); when a single colony type was found, at least three colonies were selected. Each of these colonies was grown on a TSA, KIA and in peptone water. The latter was used to standardize the innoculum of each isolate and grow them in different biochemical test media mainly: KIA, Dul, Suc, PA, indol, H_2_S, U, LCD, and ONPG (Table 1). Cells from single colonies were stored, and used to found the next selection round which was repeated throughout all 10 selective rounds (Figure 1). Biochemical variations were observed and scored (unpublished data).

All of the isolates deriving from this process were labelled and stored on sealed Dorset egg slant at a controlled room temperature along with the FMU culture collection at the Facultad de Medicina de la Universidad Nacional Autónoma de México (UNAM).

Reports concerning such variable biochemical behaviour of *C. freundii* [7-10], as well as results from the previously study, indicated that important phenotypic changes were produced. However, genetic differences were not established, leading to studies being carried out at a molecular level to understand this phenomenon.

Later in 1992 at the National Institute of Pediatrics (INP), Multi-Locus Enzyme Electrophoresis **(**MLEE**)** was performed in 302 isolates arising from the study carried out by Dr. Vázquez. The *C. freundii* strain E9750 NCTC, from the UK was used as a control. MLEE was one of only a few procedures that allowed genomic differences to be established through the identification of the chromosomal loci of basic metabolic enzymes. The main advantage of this technique was that variation in the electrophoretic mobility of enzymes could be directly related to the allelic variation in specific genes that code for the studied proteins [112].

In general, the results showed only 3 different MLEE genotypes among 302 selected isolates and the control *C. freundii* E9750. These results also highlighted that the FMU isolates that presented variations in their biochemical phenotype also presented changes in mobility in some enzymes studied, dividing the samples into two MLEE genotypes only. However, this technique was not able to determine intermediate MLEE genotypes, as observed when conventional biochemical tests are used. The results also presented changes in 15 out of 20 enzymes between *C. freundii* E9750 and atypical *C. freundii* FMU108327/A_10_, and 6 out of 20 enzymes between *C. freundii* E9750 and *C. freundii* FMU108327/P respectively. These results once again demonstrated phenotypic variability, but in this case at a molecular level (unpublished data). MLEE results allowed changes in the loci to be inferred, however this information was limited and it was not possible to relate the genetic events to phenotypic changes found in the atypical *C. freundii* strains.
